# Findings and Guidelines on Provider Technology, Fatigue, and Well-being: Scoping Review

**DOI:** 10.2196/34451

**Published:** 2022-05-25

**Authors:** Donald M Hilty, Christina M Armstrong, Shelby A Smout, Allison Crawford, Marlene M Maheu, Kenneth P Drude, Steven Chan, Peter M Yellowlees, Elizabeth A Krupinski

**Affiliations:** 1 Department of Psychiatry & Behavioral Sciences University of California Davis School of Medicine Sacramento, CA United States; 2 Northern California Veterans Affairs Health Care System Mather, CA United States; 3 Office of Connected Care, Department of Veterans Affairs Washington, DC United States; 4 Virginia Commonwealth University Richmond, VA United States; 5 Extension for Community Healthcare Outcomes, Ontario Mental Health at Centre for Addiction and Mental Health, University of Toronto Virtual Mental Health, and Canada Suicide Prevention Service Toronto, ON Canada; 6 Telebehavioral Health Institute, LLC and Coalition for Technology in Behavioral Science San Diego, CA United States; 7 Coalition for Technology in Behavioral Science & Ohio Board of Psychology Dayton, OH United States; 8 Department of Psychiatry & Behavioral Sciences Stanford University School of Medicine & Veterans Affairs Palo Alto Health Care System Palo Alto, CA United States; 9 Department of Radiology & Imaging Sciences Emory University School of Medicine Atlanta, GA United States

**Keywords:** burnout, screen fatigue, technology fatigue, well-being, videoconferencing, Zoom fatigue, mobile phone

## Abstract

**Background:**

Video and other technologies are reshaping the delivery of health care, yet barriers related to workflow and possible provider fatigue suggest that a thorough evaluation is needed for quality and process improvement.

**Objective:**

This scoping review explored the relationship among technology, fatigue, and health care to improve the conditions for providers.

**Methods:**

A 6-stage scoping review of literature (from 10 databases) published from 2000 to 2020 that focused on technology, health care, and fatigue was conducted. Technologies included synchronous video, telephone, informatics systems, asynchronous wearable sensors, and mobile health devices for health care in 4 concept areas related to provider experience: behavioral, cognitive, emotional, and physical impact; workplace at the individual, clinic, hospital, and system or organizational levels; well-being, burnout, and stress; and perceptions regarding technology. Qualitative content, discourse, and framework analyses were used to thematically analyze data for developing a spectrum of health to risk of fatigue to manifestations of burnout.

**Results:**

Of the 4221 potential literature references, 202 (4.79%) were duplicates, and our review of the titles and abstracts of 4019 (95.21%) found that 3837 (90.9%) were irrelevant. A full-text review of 182 studies revealed that 12 (6.6%) studies met all the criteria related to technology, health care, and fatigue, and these studied the behavioral, emotional, cognitive, and physical impact of workflow at the individual, hospital, and system or organizational levels. Video and electronic health record use has been associated with physical eye fatigue; neck pain; stress; tiredness; and behavioral impacts related to additional effort owing to barriers, trouble with engagement, emotional wear and tear and exhaustion, cognitive inattention, effort, expecting problems, multitasking and workload, and emotional experiences (eg, anger, irritability, stress, and concern about well-being). An additional 14 studies that evaluated behavioral, emotional, and cognitive impacts without focusing on fatigue found high user ratings on data quality, accuracy, and processing but low satisfaction with clerical tasks, the effort required in work, and interruptions costing time, resulting in more errors, stress, and frustration. Our qualitative analysis suggests a spectrum from health to risk and provides an outline of organizational approaches to human factors and technology in health care. Business, occupational health, human factors, and well-being literature have not studied technology fatigue and burnout; however, their findings help contextualize technology-based fatigue to suggest guidelines. Few studies were found to contextually evaluate differences according to health professions and practice contexts.

**Conclusions:**

Health care systems need to evaluate the impact of technology in accordance with the Quadruple Aim to support providers’ well-being and prevent workload burden, fatigue, and burnout. Implementation and effectiveness approaches and a multilevel approach with objective measures for clinical, human factors, training, professional development, and administrative workflow are suggested. This requires institutional strategies and competencies to integrate health care quality, technology and well-being outcomes.

## Introduction

### Background

Technology is reshaping the delivery of health care worldwide as a facilitator, practice extender, and virtual team member for person- and patient-centered care [1,2]. Health care systems and governmental agencies worldwide are promoting quality and evidence-based care by implementing the Quadruple Aim, which emphasizes reducing costs and improving population health, patient experience, and team well-being [1-3]. Fatigue is a very complex and multidimensional construct and a review of research across cognitive science, exercise physiology, and clinical practice suggests that its most promising common feature is the notion of *perceived effort*—this accounts for interindividual differences and situational variations and includes both mental and physical constructs and integrates motivational and emotional dimensions [4]. Health care providers and other employees have increasingly noted problems related to fatigue and excessive workflow steps, particularly electronic health records (EHRs), that may affect well-being and contribute to burnout [5].

Technology challenges include learning to use it personally and professionally and integrating it into workflow and screen time [6-8]. Subjective phrases such as *technology fatigue* or, in the COVID-19 era, *Zoom fatigue* suggest that technology *causes* fatigue. Past research on employees’ subjective, physical, and ophthalmologic factors related to computer displays [9-14] suggests that there are many additional occupational health factors related to fatigue, burnout, and accidents [15-17]. Business industries have contended with technological challenges and systematically changed workflows for users to transform and avoid extinction [18]. In health care, there appears to be a gap between the system’s perception of processes and users’ or participants’ experiences [2].

### Current Practice

It appears that there is a gap in how health care providers typically use EHRs and other technologies, the amount of effort required for workflow, and how this leads to fatigue (or burnout). Health care is starting to evaluate the longitudinal continua of work engagement and burnout, the development of burnout in relation to job demands and resources, and the role of psychosocial working conditions [19,20]. Assessment of well-being [21,22], burnout [23-28], burnout with EHRs [29,30], and related risk factors [31] is underway, including in psychiatry and behavioral health providers [32-34]. Interventions can help prevent and ameliorate burnout [35,36] and changes to organizational structure (eg, shared leadership), process improvement (eg, lean), employee support (eg, leisure, fitness, and diet), and professional development [37,38]. Another gap is that systems have generally approached burnout as an individual’s problem (eg, depression) rather than as an organization’s problem (ie, a shared problem). Key stressors within an organization that put people at risk of burnout need to be identified—at a department or unit level—so that changes can be made to reduce their impact and create healthier workplaces.

### Objective

The relationship among technology, fatigue, and health care can be better understood by reviewing the broad literature on health, business, occupational health, technology, and well-being. This will help with the following:

1. Find data on the intersection of technology, fatigue, and health care (eg, association, mediation, and cause).
2. Provide an overview of the business, occupational health, and well-being literature to contextualize technology-based fatigue, its components, and related processes.
3. Suggest guidelines for health care related to technology, well-being, and fatigue at provider, clinic, and system levels to advance self-assessment, quality improvement, and necessary organizational and social improvements to promote a culture of well-being.

## Methods

### Approach

A literature search via the Medical Subject Headings of the keywords spanned from January 2000 to December 2020, using the original 6-stage scoping review process [39], with updated modifications [40] and the PRISMA-ScR (Preferred Reporting Items for Systematic Reviews and Meta-Analyses extension for Scoping Reviews) [41].

### Research Question

This scoping review explores the relationship among technology, fatigue, and health care to improve the conditions for providers. It focuses on the overarching question: “What is technology-based fatigue and what are its consequences for providers and patients?” The subquestions are as follows:

1. What are the characteristics of technology-based fatigue and its associated factors, including technologies?
2. Does technology and associated fatigue impact provider health (burnout, compassion fatigue, and well-being)?
3. How does provider burnout or well-being associated with technology affect the delivery of care; therapeutic relationships; and quality of care offered in person, by video, and by other technologies?
4. What are strategies or interventions being used to prevent or ameliorate technology fatigue?

The goal was to synthesize clinical, provider, administrative, business, and other workplace data and consider the current and target states for using technologies in a healthy way to prevent or minimize problems and focus efforts on further assessment and intervention.

### Identifying Relevant Studies

A total of 10 literature databases were queried by a librarian: PubMed, APA PsycNET, Embase, PsycINFO database via the Ebsco platform, Web of Science, Scopus, Social Sciences Citation Index, Telemedicine Information Exchange database, Centre for Reviews and Dissemination, and Cochrane Database of Systematic Reviews and Central Register of Controlled Trials. The search focused on technology, health care, and fatigue via synchronous telepsychiatry and tele-behavioral or tele-mental health, though telephone, asynchronous, mobile health, tablets, and text were also searched. It also included types of health providers (ie, clinician, provider, counselor, employee, medical nurse or physician, psychiatrist, psychologist, social worker, therapist, and worker), assessment, care, evaluation, screening, therapy, triage, and treatment. The initial search targeted 4 concept areas by using specific terms as shown in Textbox 1.

**Concept areas used in the initial search.**

**Behavioral, cognitive, emotional, and physical impact**

- Behavioral impact
  (anxi*, barriers, boredom, complain*, concern*, depression *, detachment *, distance, effort*, engage*, emotional*, enjoy*, exhaustion, experience, factor, fatigue*, insomnia, intimacy, isolation, mental, onerous, positive, readiness, reward*, social, substance, suicide, team, worry) or
- Cognitive impact
  (attention, attitude*, alertness, critical, cynicism, distraction, efficacy, effort, expectation, incompetence, indecision, motivation, multitasking, negative, step*, task*, workflow, workload) or
- Emotional impact
  (alone, anger, anxiety, compassion*, complex, confidence, empower, esteem, human, irritability, lonely, positive, quality of life, resilien*, sadness, satisfaction, secondary, share*, trauma, satisfaction, stress, support, susceptible, therapeutic, wellness, well-being) or
- Physical impact
(ache, back, distress, exhaustion, eye, fatigue, headache, neck, pain, problem*, strain, stress, tiredness, visual)

**Workplace at the individual, clinic, hospital, and system or organizational levels**

- accessories, alternative, burden, clerical, computer, control, dedicated, demand, display, distraction, disrupti*, error*, flexib*, home, interruptions, intrusion, job, mishap, mistake, nap, organization, recognition, routine, relative value unit (RVU), safety, schedule, screen, separation, shift, telework, terminal, time, video, voice, workflow, and workload

**Well-being, burnout, and stress**

- adaptable, adjustment, burnout, confidence, cop*, esteem, fitness, happy, health*, mindful*, purposeful, relaxation, resilien*, risk, safety, satisfaction, vitality, vulnerab*, wellness, willingness

**Provider perceptions regarding technology**

- *attitudes, diffusion, adaptor, and willingness, motivation, urgency, readiness to use technology, biases regarding tech use, and experience of using technology*

### Study Selection

**Figure 1:**
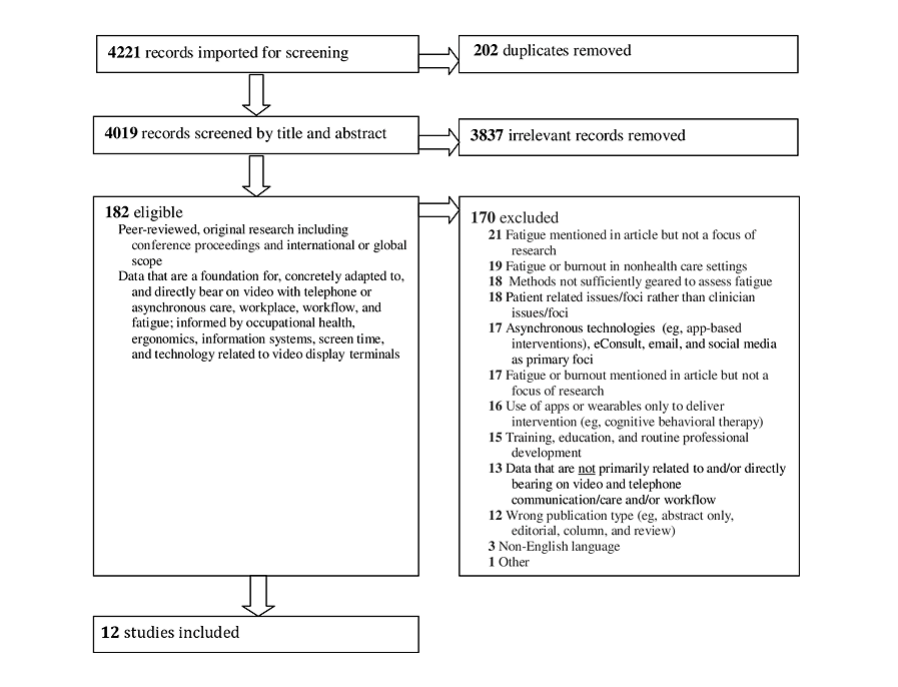
Search flowchart: diagram of studies reviewed. eConsult: electronic consultation.

One author (DMH) screened titles and abstracts of 4221 potential references, excluding 202 (4.79%) based on duplication and 3837 (90.9%) that did not meet the search criteria. Notably, 2 of 3 authors (DMH, CMA, or SAS) reviewed the full text of 182 articles, but only 12 (6.6%) met the inclusion criteria related to health care, technology, and fatigue based on consensus (Figure 1). If there was a disagreement, a third author (DMH, CMA, or SAS) made the decision. An additional 14 studies evaluated health care and technology workflow with user experiences and perceptions that may provide a contextual understanding of fatigue.

### Data Charting

A data charting form was used to extract data, and notes were organized consistent with a descriptive analytical method by each reviewer. The reviewers compared and consolidated the information using a qualitative content analysis approach [42].

### Analysis, Reporting, and the Meaning of Findings

Results were organized based on the objectives into tables and figures, with key concepts and components of technology-based fatigue outlined and described, partially based on excerpts from published topics. As this research area, although critical, is nascent, findings were reported individually.

**Figure 2:**
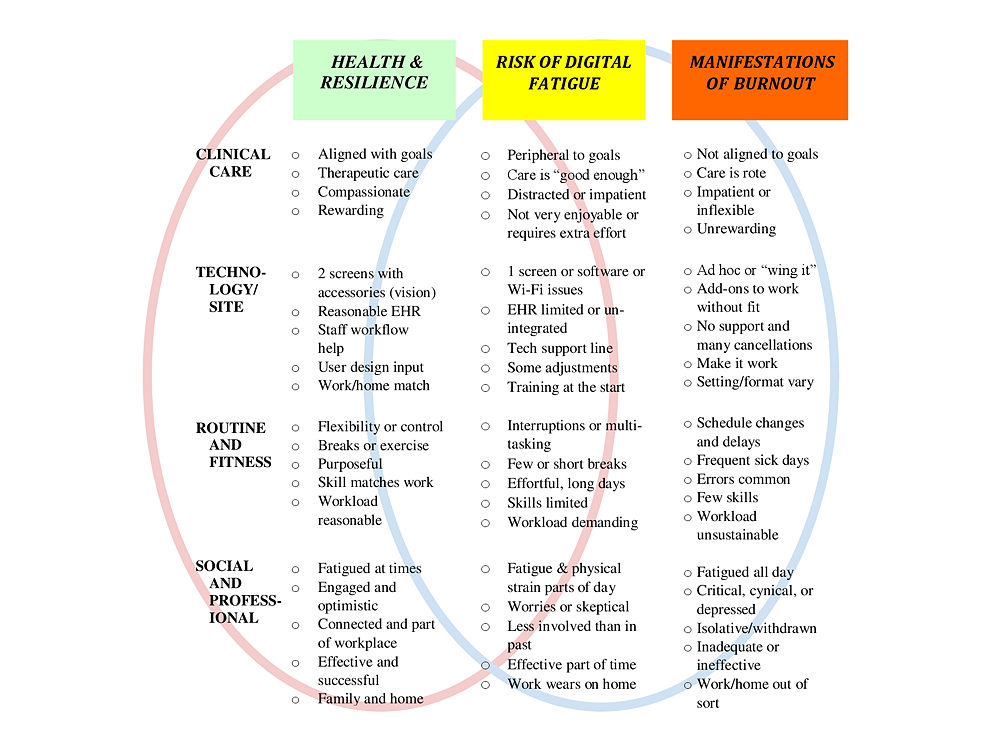
A comparison of health and resilience, risk to well-being, and manifestations of technology-based fatigue and burnout. EHR: electronic health record.

**Figure 3:**
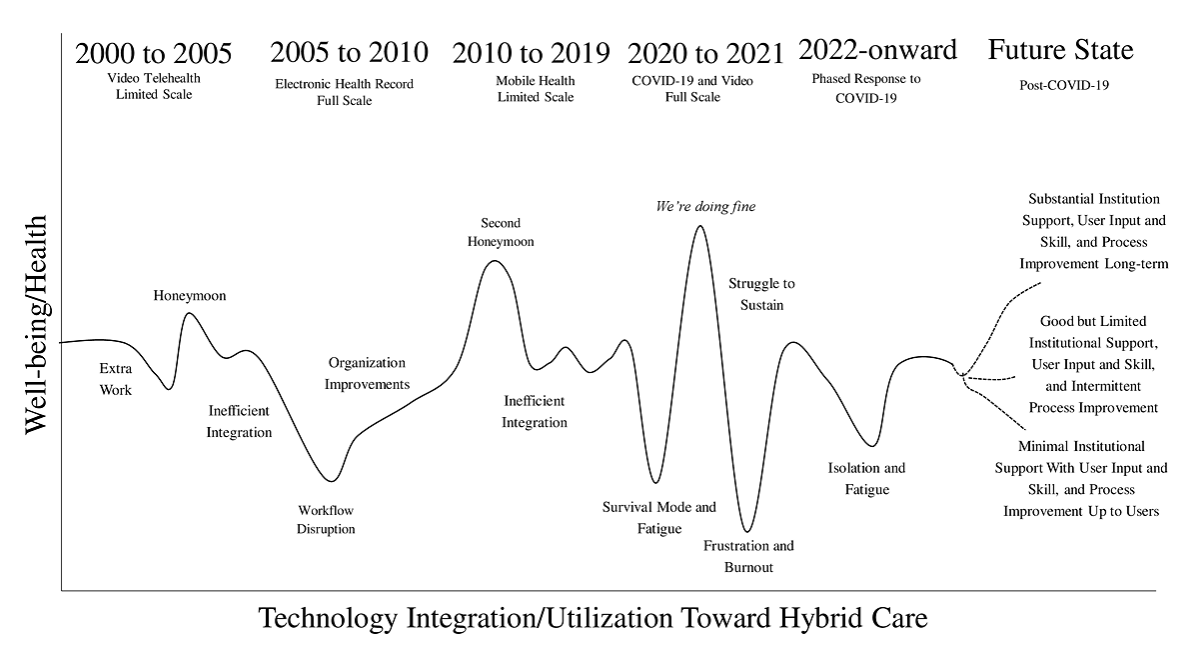
Impact of the implementation of technology integration and utilization toward hybrid care on health and well-being over time.

The technologies used have evolved considerably, making these articles a challenge to compare. Qualitative steps to analyze disparate populations, data and methods of studies were used [42]. Content, discourse, and framework qualitative analysis techniques were used to analyze findings from papers to develop a spectrum of health to risk of fatigue to manifestations of burnout (Figure 2) [43]. Content analysis was used to classify, summarize, and tabulate the behavioral data; discourse analysis was used to search for themes and patterns; and framework analysis was used to sift through, chart, and sort data in accordance with key issues and themes in a series of steps (eg, indexing, charting, mapping, and interpretation). Time points related to the release and integration of new technologies into the marketplace and health care, as well as concept area terminology surfacing in the literature were estimated qualitatively (Figure 3).

### Expert Opinions

Expert opinions were solicited to review preliminary findings and suggest additional steps for improvement. A list of relevant experts was compiled from (1) behavioral health organizations internationally (eg, Psychiatry, Psychology, Social work, and Addiction); (2) technology-related special interest groups of organizations (eg, the American Telemedicine Association American Medical or Nursing Informatics Associations and Coalition for Technology in Behavioral Sciences); (3) health organizations related to quality improvement, human resources, occupational health, and lean systems (eg, Agency for Healthcare Research and Quality, American National Standards Institute, Healthcare Information and Management Systems Society, Joint Commission, and World Health Organization); and (4) federal (ie, US National Academy of Sciences, US. National Institute of Health, US Department of Defense, and US Veterans Health Administration) and academic institutions (ie, Mayo Clinic and University of California); and (5) researchers, authors, editors, and editorial board members.

Experts were invited by email from 7 countries (Australia, Canada, Germany, India, Italy, the United Kingdom, and the United States) by several means, including attending a live videoconference expert feedback session and providing qualitative feedback. The lead author (DMH) facilitated the use of a scribe, and each of the 3 sessions lasted 50 minutes. The abstract, objectives, methods, tables, and figures were sent to experts a week in advance. The session started with a brief introduction based on the abstract, objectives, and overview of the table and figure content (10 minutes). This was followed by general questions, comments, and suggestions, including review of the data charting and search criteria (20 minutes). The input was summarized and themes were extracted to guide the organization (eg, headings in rows) and content (eg, in the columns) of tables and figures. The questions were asked to solicit additional feedback (10 minutes), and other suggestions were provided at the end of the session. Feedback was collated based on previous studies using consensus and modified Delphi processes [37,44]. Attendees were asked to complete a qualitative and quantitative 5-item Likert-scale survey (strongly disagree, disagree, neutral, agree, or strongly agree) or provide qualitative feedback via email. The survey included 6 questions, 3 weighted positively (the data provide a systematic way for clinicians to assess fatigue and well-being) and 3 weighted negatively.

## Results

### Overview

The results are organized per objectives (intersection of technology, fatigue, and health care; business, occupational health, and well-being literature; and guidelines for health care), which align with the search of the 4 concept areas (behavioral, cognitive, emotional, and physical impact; workplace at the individual, clinic, hospital, and system or organizational levels; well-being, burnout, and stress; and provider perceptions regarding technology). Business, occupational health, and well-being literature did not study technology fatigue and burnout; however, findings help contextualize technology-based fatigue to suggest guidelines at provider, clinic, and system levels for health care. Few studies were found to contextually evaluate differences according to health professions and practice contexts.

Expert opinions and feedback (N=19) contributed by attending a live expert feedback session and providing qualitative feedback, completing a qualitative and quantitative 6-item Likert-scale survey (16/19, 84%), or providing qualitative feedback via email (2/19, 10%). Of the 19 attendees in expert feedback sessions, 8 (42%) were psychiatrists, 5 (26%) were psychologists, 2 (10%) were marriage and family therapists, 1 (5%) was a physician (not psychiatrist), 1 (5%) was a counselor, 1 (5%) was a social worker, and 1 (5%) was a systems engineer. Results showed that most attendees agreed or strongly agreed that (1) “The results provided in tables are organized in the ballpark and relatively complete” (18/19 93%), (2) “The tables are a practical way to identify, analyze, and begin to address technology problems for providers and systems” (13/19, 69%); and (3) “The figures substantially help to compare and contrast the continuum of health versus fatigue versus burnout” (13/19, 69%).

### Technology, Health Care, and Fatigue

A total of 12 papers met the inclusion criteria based on the consensus of the authors [8,13,14,45-53]. Studies assessed the behavioral (8/12, 67%), emotional (4/12, 33%), cognitive (7/12, 58%), and physical (4/12, 33%) impact of workflow at the individual (11/12, 92%), clinic (8/12, 67%), hospital (6/12, 50%), and system or organizational (6/12, 50%) levels; only 25% (3/12) of studies included all levels. Most health care professionals had medical degrees (MD; 8/12, 67%), including radiologists (2/12, 17%). Video and EHR use was associated with behavioral, cognitive, emotional, and physical impact, with the latter usually reported as eye fatigue, neck pain, stress, and tiredness. Behavioral impact involved additional efforts regarding barriers, trouble with engagement, emotional wear and tear, exhaustion, and fatigue. Cognitive impact focused on inattention, effort, expecting problems, multitasking, and workload. Emotional impact was related to anger, irritability, stress, and concern about well-being.

These studies were conducted in the United States, although a study compared providers’ impact across countries. Only 17% (2/12) studies discussed the physical environment, occupational health approaches, mobile care, telework, or lean, human factors, and user design approaches to workflow. System onboarding and training enables users to get oriented and informally sets expectations, but often there are no processes for ongoing self-, peer-, and system-assessment of experience or skills. Workplace, workspace, ergonomic, and technology implementation are gaining more attention in health care [54,55] and other industries for those who function at work and home [56]. The studies were unidirectional in association, mediation, and causation—technology causing fatigue, and similar to other studies in the literature [11,12,57,58], they lacked standard assessment, monitoring, and interventions.

Studies have focused on the use [8,45,53], surveys of providers [46,51], visual strain or fatigue [13,14], implementation and usability [47,52], and consensus reports [59,60] (Multimedia Appendix 1 [8,13,14,45-53]). It is organized by study, sample size (N), length of time, population, country, design, type of technology, area of focus of the assessment (ie, behavioral, cognitive, emotional, and physical impact), and level of the assessment (ie, individual, clinic, hospital, and system or organizational). Physician participants experienced physiological fatigue at least once during simulation exercises involving 4 patient cases, with the majority (20/25, 80%) experiencing physiological fatigue within the first 22 minutes of use [8]. Those who experienced EHR-related fatigue in a patient case were less efficient in the subsequent case as demonstrated by longer task completion times (*r*=−0.521; *P*=.007), higher numbers of mouse clicks (*r*=−0.562; *P*=.003), and more EHR screen visits (*r*=−0.486; *P*=.01). Visual strain and fatigue studies have focused on individual-level adjustments for accommodation at near distances, with lack of energy, physical discomfort, and sleepiness, were statistically significantly higher as functions of the length of session [13,14]. Thus, shifts at the workplace and organizational levels may be required for the overall workflow.

Approximately 45.8% (3338/7279) of the physicians worked for >60 hours per week compared with 10% (3442/34,420) of US workers in other ﬁelds [51]. Studies have determined that physicians spend 4 to 6 hours on EHR and desk work during the day and another 1 to 2 hours after work, often for clerical and administrative tasks (eg, documentation, order entry, billing, coding, and system security) [52]. Studies found that US providers compared with others spent substantially more time actively using the EHR (mean time 90.2 minutes vs 59.1 minutes; *P*<.001), including making notes, orders, in-basket messages, and clinical review [45]. They also composed more automated note text than their non-US counterparts (270/348, 77.5% vs 14/23, 61%; *P*<.001) and received statistically significantly more messages per day (33.8 vs 12.8; *P*<.001). Furthermore, US clinicians used the EHR for a longer time after hours, logging in 26.5 minutes per day versus 19.5 minutes per day for non-US clinicians (*P*=.01). These results persisted after controlling for organizational characteristics, including structure, type, size, and daily patient volume. The most important 3 factors that separate the ideal order sets from the rest are patient safety, efficiency, and user satisfaction. Scientific evidence, workflow, ordering efficiency, and user satisfaction reduce mouse clicks and unproductive thinking times [53].

Implementation studies of usability suggest that there are multiple opportunities to improve the use of EHRs across professions, particularly in relation to usability [47,51-53]. A survey on health information technology (IT) for pharmacy practice showed that some EHRs may also introduce new error types (eg, excessive alerts can lead to fatigue, so much so that providers can inadvertently ignore scanner barcode indicators of drug mismatches and erroneously identify drugs) [46].

### Provider Perceptions and Experiences With Technology in Health Care Not Specific to Fatigue

A total of 14 studies explored provider experiences or perceptions about technology that may apply to fatigue but did not directly investigate it. These studies focused on EHR and videos (6/14, 43%); combinations of video display terminals (VDTs), computers, and phones (6/14, 43%); smartphones or PDA (1/14, 7%), or EHR alone (1/14, 7%; Multimedia Appendix 2 [6,56,61-72]). Methods were heterogeneous with surveys, semistructured interviews, qualitative methods, and comparison groups (eg, video vs in-person or other). The foci of the assessment were behavioral (9/14, 64%), emotional (9/14, 64%), cognitive (10/14, 71%), and physical impact (1/14, 7%) and the assessment was at the individual (12/14, 86%), clinic (6/14, 43%), hospital (6/14, 43%), and system or organizational (6/14, 43%) levels.

Studies that focused on EHR time log data for physicians [61,71] found substantial time of EHR use (eg, 5.9 hours of a 11.4-hour workday in a hospital, 4.5 hours during clinics hours, and 1.4 hours after clinic). Of time spent in the EHR, clerical and administrative tasks such as documentation, order entry, billing, and coding accounted for 44.2%, inbox management for 23.7%, and additional time communicating with patients, refilling prescriptions, or reviewing test results each day [61,71]. User ratings were high on data quality, accuracy, and processing [62] but low for satisfaction with clerical tasks [6]. Interrupted tasks require more time and result in more errors, stress, and frustration [72], and qualitative interviews and focus groups suggested more focus on usability, usefulness, training, and support [65,68]. There were differences among generations regarding adaptability, perceived benefits and drawbacks, and perceptions of other generations’ abilities to adapt.

### A Continuum From Health to Fatigue to Burnout

Qualitative analysis of the literature suggests a continuum from health to risk of fatigue to manifestations of digital burnout (Figure 2). This was stratified by clinical care, technology, routine, and social, interpersonal and professional dimensions. Related to care, providers vary in how aligned technology is with goals, how *therapeutic* or enjoyable it is for them (and not just patients), and other rewards. Organizations play a substantial role in selection and implementation of technology for clinical workflow, workload, and remuneration, which often predetermine routines. Provider input is sometimes solicited. When organizations use user-centered design or lean processes, user satisfaction and the fit of goals, methods, and routines may be much higher [59,60], avoiding gaps between the system and provider perspectives. Fatigue often manifests in social and interpersonal contexts, with taxing fatigue overtaking effectiveness and engagement, resulting in burnout with exhaustion, cynicism, and feelings of ineffectiveness [6,24,25].

### Organizational Responses Based on Provider Experiences and Human Factors Related to Technology

A qualitative analysis suggested multiple opportunities for regulators, policy makers, EHR developers, payers, health system leadership, and users to collectively improve the use of EHRs and other technologies (Multimedia Appendix 3) [47,52]. It summarizes human factors and technology in health care: organizational responses for prevention and adjustment of workflow, as organized in terms of evidence and findings, manifestations and analysis of technology problems, and individual user versus organizational adjustments being made. A change management process for workflow and administration [73] requires training, supervision, and evaluation to adjust competencies or skills, improve quality and performance indicators, and reallocate resources in health care [2,74].

### Business, Occupational Health, and Well-being Literature to Contextualize Technology-Based Fatigue

#### Overview

From the 1980s to 2021, there has been a shift in the perception of the origin of technology problems in business, occupational health, and other area [46,75]. Earlier perceptions attributed problems of production, efficiency, and outputs as being related to ergonomic, mechanical, workplace, and other factors for individuals, cohorts (eg, VDT employees), and systems. More recently, gaining input from users of technology is central to the design of the workplace to minimize and prevent problems.

#### VDT Studies

Findings from VDT studies of occupational hygiene (eg, climate, lighting, and electrostatic conditions) and ophthalmologic dimensions appear to be quite pertinent to video, EHR, and psychosocial work commonly associated with *technology fatigue* [9,10,76]. Job stress has been found to be higher; quality of life has been found to be lower; and visual strain, discomfort, and fatigue has been found to be higher in VDT workers than in non-VDT workers [77], and combined, interactive communication causes more discomfort than data entry or acquisition. Postural risk factors and job strain in the environment seem to increase musculoskeletal symptoms for those with >7 hours of VDT use per day, but ergonomic interventions improve function [57,78].

Displays and workflow interventions have been successful in many respects. A 15-minute work period with microbreaks [79] and physically large displays help improve employee performance. A 15° rather than 40° video display curvature (display curvature impacts effort to visualize displayed text) [80] and a case manuscript and luminance ratio of 3 (used for the useful contrast of a display) also help users’ performance [9-14]. Coworking spaces are an alternative to home offices because professional isolation negatively affects job performance, and employees with inhibitory deficits (eg, prone to distraction) and poorer boundaries (ie, limited psychological detachment) experience more stress [58,81].

#### Studies Assessing Fatigue and Burnout

There are overlaps and differences between burnout and prolonged fatigue [82]. Fatigue plays a central role in the development of burnout (ie, medical) and prolonged fatigue (ie, Psychological), with the former conceptualized as a work-related condition and prolonged fatigue as a general condition. Burnout manifests as exhaustion (physical and emotional), cynicism and detachment from the job and others, and a sense of ineffectiveness and lack of accomplishment [24,25]. Low job dissatisfaction is associated with low organizational commitment, absenteeism, intention to leave the job, turnover, lower productivity, and impaired quality of work. Those who experience burnout also disrupt job tasks and display greater interpersonal aggression [24,25]. The Areas of Worklife model considers workload, sense of control, reward, community, fairness, and values as important to burnout [75]. Rewards and recognition provide opportunities for intrinsic satisfaction and self-efficacy. A good community provides social support, trust, effective means of working out disagreement, and better job engagement. The job demands-resources model posits that burnout is related to the experience of incessant job demands and inadequate resources, whereas the conservation of resources model follows basic motivational theory in assuming that burnout arises because of persistent threats to available resources.

Conceptualization of fatigue and burnout may also be organized according to engagement and job stress [24,25,83]. Engagement is considered a state of high energy, strong involvement, and a sense of efficacy. It is a persistent, positive, affective-motivational state of fulfillment characterized by the 3 components of vigor, dedication, and absorption. Engagement is considered an independent and distinct concept, which is not the opposite of burnout, although is negatively related to it. Interventions at the *individual level* may involve the following: (1) changing work patterns, (2) developing coping skills, (3) obtaining social support, (4) using relaxation strategies, (5) promoting good health and fitness, and (6) developing a better self-understanding. At the *workplace or organizational level*, this may mean the following: (1) redesigning job tasks, (2) improving recognition of notable work by both teams and individuals, and (3) developing more fair and equitable policies.

### Guidelines for Providers, Systems, and Organizations in Health Care for Use of Technology and Well-being

A shift to a culture of well-being with technology use requires the evaluation, implementation, and monitoring of individual, workplace, workflow, and institutional strategies (Multimedia Appendix 4 [2,6,7,23-25,28-30,37,44,49-53,56,74,83-85]). If the link between technology and fatigue is poorly recognized, changes in workflow processes and policies may not be carried out until the provider’s well-being is already at risk owing to burnout [49]. Guidelines for health care, well-being, and the use of technology (eg, EHRs) to avoid burnout were found throughout these studies and summarized as well; however, these need to evaluate fatigue.

A shift to a culture of well-being that incorporates technology will require adaptations and quality improvement in the areas of technology, physical environment, occupational health, and specific evaluations and interventions. Therefore, objective measures to evaluate, promote, and enhance well-being are required. At a minimum, consideration is needed for the cognitive, behavioral, emotional, and physical impact of workflows. Such consideration is needed at the individual, clinic, hospital, and system or organizational levels. This could include adjustments in information systems (IS) and IT, use of lean methods and emphasis on interprofessional education efforts with technology team-based care from the Institute of Healthcare Improvement and Agency for Healthcare Research and Quality [2]. More specifically, methods are needed to evaluate clinical workflows, promote provider competencies with technology and self-care and implement institutional competencies for technologies. Deliberate, sustained, and comprehensive efforts by the organization are often inexpensive, reduce burnout, and promote engagement [24,25,49-51].

Health care provider well-being and health appear to be related to the technological integration of video, EHR, and mobile health over time (Figure 3). This figure was created based on time points related to the following: (1) the release of new technologies into the marketplace; (2) the introduction (or in some cases integration) of technologies into workflow for health care systems, which was generalized, as some systems integrated sooner and others later, and private practice providers were likely quite heterogeneously adapting; and (3) content and discourse analysis to thematically capture concept area terminology surfacing in the literature related to technology (eg, burnout has been identified much earlier, but fatigue and technology have surfaced in recent years). Organizational efforts and resultant outcomes for well-being may be stratified from high to low based on individual, system, and organizational contributions, as follows: (1) high—substantial institutional support to seek user input, optimize clinical skills, and monitor resilience and well-being; (2) mid—good but limited institutional support to include user input, which improves some workflow processes but not systematically; and (3) low—minimal institutional support with expectations that users learn, adapt workflow, and maintain well-being individually.

## Discussion

### Principal Findings

Studies related to the implementation and evaluation of technology are increasingly sophisticated and provide a starting place despite varying widely in duration, approaches, methods, and quality of measures. The 12 studies that met all the inclusion criteria for technology, health care, and fatigue studied the behavioral, emotional, cognitive, and physical impact of workflow at individual, hospital, and system or organizational levels (Multimedia Appendix 1) [8,13,14,45-53]. Video and EHR use is associated with fatigue with physical eye fatigue, neck pain, stress, and tiredness; behavioral impact related to additional effort owing to barriers, trouble with engagement, emotional wear and tear and exhaustion, cognitive inattention, effort, expecting problems, multitasking and workload, and emotional experiences such as anger, irritability, stress, and concern about well-being. An additional 14 studies that evaluated the behavioral, emotional, and cognitive impact of using technology without focusing on fatigue found high user ratings on data quality, accuracy, and processing but low satisfaction with clerical tasks, the effort required in work and interruptions costing time and resulting in more errors, stress, and frustration (Multimedia Appendix 2) [6,56,61-72]. Other problems contributing to fatigue may include the addition of workflow steps before and after clinical care is provided, often requiring sustained periods of technology use. Few studies have discussed the physical environment, occupational health approaches, mobile, telework, lean, process improvement, human factors, and user design approaches to workflows. A qualitative analysis of the literature suggests a continuum from health to risk of fatigue to manifestations of digital burnout, which provides an outline of organization approaches to human factors and technology in health care (Figure 2). Although business, occupational health, and well-being literature did not study technology fatigue and burnout, findings from the literature help contextualize technology-based fatigue and modern approaches they use such as lean, process improvement, occupational health, design studios, and implementation science that could be helpful in health care at individual, clinic, hospital, and system or organizational levels. Few studies were found to contextually evaluate differences according to health professions and practice contexts.

Beneficial changes in workplace culture, focus on well-being, and prevention of burnout from other fields are beginning to be used in health care [29,49,86], but an accurate evaluation of the problems is just beginning. Areas of specific focus include clinical care, human factors, training, professional development, workflow, and administration factors (Multimedia Appendix 3). The conceptualization of *burnout* is undergoing change, with a shift toward the recognition of burnout as an occupational phenomenon rather than solely as an individual medical disease (eg, depression) per the World Health Organization [87]. Thus, deployment of health care and administrative resources should move beyond the individual (eg, Family and Medical Leave Act) and look at structural, educational, cultural, and social factors.

To begin to address challenges in health care related to fatigue and burnout, including those associated with technology, a substantial collaborative effort is needed from health system leadership, organizational researchers, IT and IS specialists, and potentially the government [2,3,28,29], as changes in financing, reimbursement, and regulatory processes may need adjustment. An overall approach requires implementation, evaluation, and monitoring of individual, workplace, workflow, and institutional strategies (Multimedia Appendix 4). Financial support resources (eg, counseling, retirement planning, and college planning for children) can reduce competing demands for time and address personal and career concerns [51]. To transform organizational culture, *wrap-around* support for providers, not just patients, may be needed, as suggested by the Quadruple Aim. In the business culture of successful companies, such as Cirque du Soleil, L’Oreal Paris, and Nintendo, the tetrad foci of research, production, marketing, and finance have been expanded to a pentad by integrating technology rather than appending it [18,38,88]. An IT business–medicine understanding or conceptual framework has likewise been suggested with individual and institutional competencies [2,74] based on IT architecture [84].

A structural and functional redesign of systems would emphasize evaluation, effectiveness, implementation, and application of process improvement [59,60,85,89]. It includes approaches to causal questions using cross-sectional and longitudinal dimensions, multilevel foci, and objective measures for clinical (engagement, meaningfulness of tasks, process and quality measures, and clinical and safety outcomes), human factors (workload, rewards, fitness, needs, and well-being), training, professional development, and administrative (value alignment, productivity, IS, strategic planning, resources, and participative management) workflows. A 360° perspective with qualitative approaches could be useful to collect input from providers on what makes care *therapeutic*, enjoyable and easy to provide, and promotes their well-being and performance as individuals, team members, and leaders. In time, continuous data collection and analytics could support clinical decision-making for patient quality, workforce satisfaction, and system outcomes, creating an organizational culture of well-being, compassion in care, and prevention of fatigue and burnout in all employees, including providers [2,3,90]. Human factors engineering and usability assessment has a rich set of scientiﬁc methods, a strong evidence base, and is widely applied effectively in other industries [84,85].

### Limitations

This scoping review has some limitations. First, there were fewer findings than we expected using our inclusion and exclusion criteria, despite a broad scope, to find the relationships between health care, fatigue, and synchronous (video, telephone, and informatic systems) and asynchronous (wearable sensors and mobile health devices) technologies. Second, only 1 author reviewed the titles and abstracts. Third, the entire search was described but not saved and consolidated as an appendix for reviewers; reresearch findings of the 2 main databases were included as an appendix for reviewers. Although the terms, databases, and dates are a guide to other researchers, this omission does not enable others to simulate the approach. Fourth, given the small sample sizes, heterogeneous methods, and variable study duration, the team was unable to apply a systematic quality evaluation system or draw conclusions using a quantitative meta-analysis. Cross-sectional studies of associations with multiple factors in applied rather than controlled settings have limitations. Fifth, the stratification of behavioral, cognitive, emotional, and physical domains of impact, although heuristically helpful, could have been operationalized more rigorously. Similarly, *workplace* at the individual, clinic, hospital, and system or organizational levels may need better definitions. Sixth, the review does not cover all potentially relevant well-being, burnout, and stress dimensions of the workplace, nor does it cover research on the physical environment, occupational health, or mobile, virtual or telework workflows. Seventh, broader input for consensus across organizations could have been helpful, and a qualitative, small group interview approach with experts using a semistructured guide could have discovered more information.

### Conclusions

Health care delivery and systems are increasingly incorporating technology but need to evaluate its impact in accordance with the Quadruple Aim to support providers. Approaches with causal questions and longitudinal implementation research could benefit from a multilevel approach with objective measures for clinical and human factors, training, professional development, and administrative workflows. If done well, technology integration could further population-centered health and effectiveness of service delivery, although the redesign of financing, reimbursement, regulatory, and other changes may be necessary. Integration of health care quality outcomes with those for technology and well-being is suggested and requires institutional strategies and competencies. Otherwise, continued advances in the use of technology may inadvertently worsen provider workload burden, fatigue, and burnout.

## Appendix

Multimedia Appendix 1 Studies at the intersection of technology, health care, and fatigue.

Multimedia Appendix 2 Studies of the reviews focused on technology-related experiences in health care for clinicians aside from fatigue.

Multimedia Appendix 3 Human factors and technology in health care: organizational responses for prevention and adjustment of workflow.

Multimedia Appendix 4 Guidelines for clinicians, systems, and organizations in health care for technology use and well-being.

## Abbreviations

EHR: electronic health record
IS: information systems
IT: information technology
PRISMA-ScR: Preferred Reporting Items for Systematic Reviews and Meta-Analyses extension for Scoping Reviews
VDT: video display terminal
