# Supplementary material for: Findings and Guidelines on Provider Technology, Fatigue, and Well-being: Scoping Review
Source: J Med Internet Res. 2022 May 25;24(5):e34451. doi: 10.2196/34451 (PMC9178447; doi:10.2196/34451)
Supplement: Multimedia Appendix 1 [file jmir_v24i5e34451_app1.docx]

**TABLE 1. STUDIES AT THE INTERSECTION OF TECHNOLOGY, HEALTH CARE AND FATIGUE.**

| **ARTICLE #** | | **STUDY** | **N** | **LENGTH (OF STUDY)** | **POPULATION** | **COUNTRY** | **DESIGN** | **METHODS** | **KEY FINDINGS** | **TECHNOLOGY (i.e. hardware, software)** | **AREA OF FOCUS*** | | | | **LEVEL OF FOCUS**** | | | |
| --- | --- | --- | --- | --- | --- | --- | --- | --- | --- | --- | --- | --- | --- | --- | --- | --- | --- | --- |
|  |  |  |  |  |  |  |  |  |  |  | (1=yes, 0=no) | | | | (1=yes, 0=no) | | | |
|  |  |  |  |  |  |  |  |  |  |  | B | E | C | P | I | C | H | S |
| **Technology, Health Care and Fatigue** | | | | | | | | | | | | | | | | | | |
| 1 | Galt et al. 2019 [46] | | 2195 | Not specified | Pharmacists | USA | Cross-sectional descriptive analytic study | Survey | Integration of health technology into pharmacy practice introduced new error types and alert fatigue | Video monitor, computer | 0 | 0 | 1 | 0 | 1 | 1 | 0 | 1 |
| 2 | Holmgren et al 2020 [45] | | N/A | 10 months | Providers | USA, Canada, Northern Europe, Western Europe, Middle East, and Oceania | Cross-sectional descriptive analytic study of full-time use | Cross-sectional; controlling for organizational characteristics, including structure, type, size, and daily patient volume. | EHR (mean time, 90.2 minutes vs 59.1 minutes; *P* < .001), including doing notes, orders, in-basket messages and clinical review. They also composed more automated note text than their non-US counterparts (77.5% vs 60.8% of note text; *P* < .001) and received statistically significantly more messages per day (33.8 vs 12.8; *P* < .001). Furthermore, US clinicians used the EHR for a longer time after hours, logging in 26.5 minutes per day vs 19.5 minutes per day for non-US clinicians (*P* = .01). | EHR, video monitor, computer | 1 | 0 | 0 | 0 | 1 | 0 | 0 | 1 |
| 3 | Khairat et al. 2020 [8] | | 25 | Not specified | Physicians | USA | Cross-sectional descriptive analytic study | EHR simulation exercise involving 4 patient cases (mean [SD] completion time, 34:43 [11:41] minutes) recorded a total of 14 hours and 27 minutes of EHR activity | Physicians: 20 of 25 participants (80%) experienced physiological fatigue within the first 22 minutes of EHR use. Physicians who experienced EHR-related fatigue in 1 patient case were less efficient in the subsequent patient case, as demonstrated by longer task completion times (*r* = −0.521; *P* = .007), higher numbers of mouse clicks (*r* = −0.562; *P* = .003), and more EHR screen visits (*r* = −0.486; *P* = .01). | EHR, video monitor, computer | 1 | 0 | 0 | 1 | 1 | 0 | 0 | 0 |
| 4 | Krupinski et al. 2009 [13] | | 6 | Not specified | Radiologists | USA | Observational study of full-time use | Use of an autorefractor to measure eyestrain at various reading distances. On average, participants had been reading CT or x-rays 5.73 hours (sd = 1.24) in late afternoon | Nearer user distance to video displays increased eyestrain. There was a statistically significant difference (F = 1188.36, p < 0.0001) in the data as a function of target distance, as a function of time of day (F = 316.10, p < 0.0001) and radiologists more than residents (F = 271.47, p < 0.0001). | Video monitor, computer, autorefractor | 0 | 0 | 0 | 1 | 1 | 0 | 0 | 0 |
| 5 | Krupinski et al. 2010 [14] | | 40 | Not specified | Radiologists | USA | Observational study full-time use | Reading time, visual accommodations, and symptoms of fatigue were measured after varied reading schedules (prior to work day and after work day) | After a day of clinical reading, radiologists have reduced ability to focus, increased symptoms of fatigue and oculomotor strain, and reduced ability to detect fractures. Lack of energy, physical discomfort and sleepiness were statistically significantly higher as a function of the length of session. | Video monitor, computer | 0 | 0 | 1 | 1 | 1 | 0 | 0 | 0 |
| 6 | Lopez et al. 2018 [47] | | N/A | Not specified | Nurses | USA | Usability study | Assessment of technology usability in healthcare can be categorized into 4 major types: inspection methods, formal usability testing, summative testing, and field studies. | Technologies are, and will be for the foreseeable future, central to the work of intensive care nurses. These technologies must be highly usable to ensure high quality and safe care delivery without unnecessary increases in workload. | Technology (general) | 1 | 0 | 1 | 0 | 1 | 1 | 1 | 0 |
| 7 | Mylod et al. 2017 [48] | | N/A | Not specified | Physicians | USA | Intervention related to full-time use | Framework | A two-pronged approach to clinician burnout is proposed whereby inherent stress is addressed separately from external stress so as to best manage the various stressors present in clinical settings that contribute to clinician burnout. | EHR, video monitor, computer | 0 | 1 | 1 | 0 | 1 | 1 | 1 | 1 |
| 8 | Nakagawa et al. 2020 [49] | | N/A | Not specified | Physicians | USA | Model intervention full-time use | Consensus | Technological advancement improve efficiency, allow more flexible working arrangements, and increase convenience for patients and physicians. | EHR, video monitor, computer + (phone, email, tablet, social media, virtual assistants) | 1 | 1 | 1 | 0 | 1 | 1 | 1 | 1 |
| 9 | National Academies of Science, 2019 [50] | | N/A | Not specified | Providers | USA | Consensus | Consensus methodology | Health care technology is always evolving and that these developments are likely to be disruptive to the care delivery system as we know it today. | EHR, video monitor, computer | 1 | 1 | 1 | 0 | 0 | 1 | 1 | 1 |
| 10 | Shanafelt et al. 2017 [51] | | N/A | Not specified | Physicians, Healthcare Administrators | USA | Assessment related to full-time use | Survey | Deliberate, sustained, and comprehensive efforts by the organization to reduce burnout and promote engagement can make a difference. Many effective interventions are relatively inexpensive, and small investments can have a large impact.  Approximately 45% of physicians work more than 60 hours per week compared with less than 10% of US workers in other ﬁelds. | Technology (general) | 1 | 1 | 0 | 0 | 1 | 1 | 1 | 1 |
| 11 | Tutty et al. 2019 [52] | | N/A | Not specified | Physicians | USA | Identification of intervention options for full-time use | Implementation | Multiple opportunities for regulators, policymakers, EHR developers, payers, health system leadership, and users to make changes collectively to improve the use of efficacy of EHRs. Studies have determined that physicians spend 4-6 hours on EHR and desk work during the day and another 1-2 hours after work, often for clerical and administrative tasks (e.g., documentation, or- der entry, billing, coding, and system security). | EHR, video monitor, computer | 1 | 0 | 0 | 1 | 1 | 1 | 1 | 1 |
| 12 | Zhang et al. 2013 [53] | | 388 | Not specified | Physicians | USA | Quasi-experimental for full-time use | Measure cognitive workload via number of clicks for different order set combinations | Excessive cognitive workload on physicians imposed by technology impacts the cognitive behavior of users. The most important three factors that separate ideal order sets from the rest are patient safety, efficiency, and user satisfaction. Scientific evidence, workflow, ordering efficiency and user satisfaction reduces mouse clicks and unproductive thinking time. | EHR, video monitor, computer | 1 | 0 | 1 | 0 | 1 | 1 | 0 | 0 |
| # of 12 | | | | | | | | | | | 8 | 4 | 7 | 4 | 11 | 8 | 6 | 6 |
| Average | | | | | | | | | | | 66.7% | 33.3% | 58.3% | 33.3% | 91.7% | 66.7% | 50.0% | 50.0% |

Acronyms: * Area of focus: B=Behavioral, E=Emotional, C=Cognitive, P=Physical; ** Level of focus: I=Individual, C=Clinic, H=Hospital, S=System; USA = United States of America, UK = United Kingdom; EHR = electronic health record, VDT = video monitor
