# Supplementary material for: Findings and Guidelines on Provider Technology, Fatigue, and Well-being: Scoping Review
Source: J Med Internet Res. 2022 May 25;24(5):e34451. doi: 10.2196/34451 (PMC9178447; doi:10.2196/34451)
Supplement: Multimedia Appendix 2 [file jmir_v24i5e34451_app2.docx]

**TABLE 2. STUDIES OF THE REVIEW FOCUSED ON TECHNOLOGY-RELATED EXPERIENCES IN HEALTH CARE FOR CLINICIANS ASIDE FROM FATIGUE.**

| **ARTICLE #** | **STUDY** | **N** | **LENGTH (of study)** | **POPULATION** | **COUNTRY** | **DESIGN** | **METHODS** | **KEY FINDINGS** | **TECHNOLOGY (i.e. hardware, software)** | **AREA OF FOCUS*** | | | | **LEVEL OF FOCUS**** | | | |
| --- | --- | --- | --- | --- | --- | --- | --- | --- | --- | --- | --- | --- | --- | --- | --- | --- | --- |
|  |  |  |  |  |  |  |  |  |  | (1=yes, 0=no) | | | | (1=yes, 0=no) | | | |
|  |  |  |  |  |  |  |  |  |  | B | E | C | P | I | C | H | S |
| 1 | Arndt et al. 2017 [61] | 142 | 3 years | Physicians | USA | Retrospective cohort study | EHR time log data | Clinicians spent 355 minutes (5.9 hours) of an 11.4-hour workday in the EHR per weekday per 1.0 clinical full-time equivalent: 269 minutes (4.5 hours) during clinic hours and 86 minutes (1.4 hours) after clinic hours | EHR, video monitor, computer, tablet | 1 | 0 | 1 | 0 | 1 | 0 | 0 | 0 |
| 2 | Campbell et al 2007 [62] | 95 | 3-4 days per site | Physicians | USA | Qualitative | Qualitative surveys and interviews with physicians | Technology can create chaos when there are insufficient backup systems in place, users have false expectations regarding data accuracy and processing, and some clinicians cannot work efficiently without computerized systems | EHR | 1 | 0 | 1 | 0 | 1 | 1 | 1 | 0 |
| 3 | Hilty et al. 2007 [63] | 94 | 3 years | Physicians, rural patients with depression | USA | Mixed methods | Qualitative and quantitative | Intensive modules using telepsychiatric educational interventions toward PCPs may be superior, but the most critical ingredient may be administrative tracking of patients | Video monitor, computer, phone | 0 | 1 | 0 | 0 | 1 | 1 | 0 | 0 |
| 4 | McAlearney et al. 2005 [64] | 161 | 6 months | Physicians | USA | Qualitative | Qualitative interviews and focus groups using standard, semi-structured guide with open-ended questions to promote discussion | Organizational strategies should include active support for broad-based and niche use, active support for niche use, and basic support for individual physicians | Smartphones, personal data assistants (PDAs) | 1 | 1 | 1 | 0 | 1 | 0 | 0 | 1 |
| 5 | McAlearney et al. 2015 [65] | 82 | Not specified | Physicians | USA | Qualitative | Qualitative interviews and focus groups to explore physician and administrative barriers to EHR: ﬁnancial, technical, time, psychological, social, legal, organizational, and change process | Recommendations provided regarding how to facilitate physician adoption and use and address organizational concerns | EHR, video monitor, computer | 1 | 1 | 1 | 0 | 1 | 1 | 1 | 1 |
| 6 | Middleton et al. 2013 [56] |  | Not specified | Healthcare professionals | USA | Consensus | 2011 Health Care Technology Foundation Clinical Alarms Survey | Provided recommendations regarding human factors health information technology research, health IT policy, industry recommendations, and recommendations for the clinician end-user of EHR software | EHR, video monitor, computer | 1 | 1 | 1 | 0 | 0 | 1 | 1 | 1 |
| 7 | Mirhafez et al. 2019 [66] | 197 | 5 months | Nurses | Iran | Cross-sectional descriptive analytical study | Surveys from nurses across three hospitals | Hospitals need to manage alarms to decrease alarm fatigue, improve alarm system safety and further develop clinical policies and procedures | EHR, video monitor, computer | 0 | 1 | 1 | 0 | 1 | 0 | 0 | 0 |
| 8 | Nakagawa et al. 2019 [67] |  | Not specified | Physicians | USA | Mixed methods | Semi-structured interviews with focus groups or individual physicians | Despite the increasing adoption of technology into medical practice, the benefits that technology can offer to physician health remain largely untapped. It is time to start evaluating the impact of health technologies on physician well-being using tools | EHR, video monitor, computer, phone, email | 1 | 1 | 1 | 0 | 1 | 0 | 0 | 1 |
| 9 | Nimjee et al. 2020 [68] | 11 | 1 month | Physicians | Canada | Qualitative | Semi-structured interviews | Common problems encountered included usability issues, downtimes, alarm fatigue, and administrative tasks. There were differences between generations regarding adaptability, perceived benefits and drawbacks and perceptions of other generations’ ability to adapt | EHR, video monitor, computer | 1 | 0 | 0 | 0 | 0 | 1 | 1 | 1 |
| 10 | Paterick et al. 2018 [69] |  | Not specified | Physicians | USA | Qualitative | Qualitative methods were used to gather and analyze data describing unintended adverse consequences of computerized provider order entry |  | EHR, video monitor, computer | 1 | 0 | 1 | 0 | 1 | 1 | 1 | 0 |
| 11 | Shanafelt et al. 2016 [6] | 6375 | 3 months | Physicians | USA | Non-experimental quantitative survey | U.S. physicians across all specialties were surveyed from August to October 2014 and provided information regarding their use to EHR, electronic patient portals, and computerized physician order entry | Physicians who used EHRs were less satisfied with the amount of time spent on clerical tasks and were at higher risk for professional burnout | EHR, video monitor, computer, electronic patient portal | 0 | 1 | 0 | 0 | 1 | 0 | 0 | 0 |
| 12 | Sockalingam et al. 2020 [70] | 426 | Not specified | Health care professionals | Canada | Implementation study | Rapid implementation and survey | Using an iterative curriculum design approach and existing implementation frameworks, the tele-education model can be rapidly mobilized to address health needs during the COVID-19 pandemic | video monitor, computer, real time polling | 0 | 1 | 0 | 0 | 1 | 0 | 0 | 0 |
| 13 | Tai-Seale et al. 2017 [71] | 471 | 3 years | Physicians, healthcare administrators | USA | Retrospective cohorts | Measure amount of time physician spent in EHR vs face to face care provided to patients | Physicians had an increase in computer related tasks and decrease in face-to-face time with patients over time | EHR, video monitor, computer, phone, email | 1 | 1 | 1 | 0 | 1 | 0 | 1 | 1 |
| 14 | Westbrook et al. 2010 [72] | 40 | Not specified | Physicians | Australia | System redesign | Observational time and motion study conducted in the 400-bed emergency department of a teaching hospital | Task completion times are shorter for interrupted tasks; tasks with high frequency of interruptions have lower accuracy; stress and frustration are higher during interrupted tasks | Personal digital assistant (PDA) | 1 | 0 | 1 | 0 | 1 | 0 | 0 | 0 |
| # out of 14 | | | | | | | | | | 9 | 9 | 10 | 1 | 12 | 6 | 6 | 6 |
| Concept Area 1a Average | | | | | | | | | | 64.3% | 64.3% | 71.4% | 7.1% | 85.7% | 42.9% | 42.9% | 46.2% |

Acronyms: * Area of focus: B=Behavioral, E=Emotional, C=Cognitive, P=Physical; ** Level of focus: I=Individual, C=Clinic, H=Hospital, S=System; USA = United States of America, UK = United Kingdom; EHR = electronic health record, PCP = primary care provider, VDT = video monitor
