# Supplementary material for: Findings and Guidelines on Provider Technology, Fatigue, and Well-being: Scoping Review
Source: J Med Internet Res. 2022 May 25;24(5):e34451. doi: 10.2196/34451 (PMC9178447; doi:10.2196/34451)
Supplement: Multimedia Appendix 3 [file jmir_v24i5e34451_app3.docx]

**TABLE 3. HUMAN FACTORS AND TECHNOLOGY IN HEALTH CARE: ORGANIZATIONAL RESPONSES FOR PREVENTION AND ADJUSTMENT OF WORKFLOW.**

**ABBREVIATIONS/DEFINITIONS/KEY:** financial = $; clinical case conference = CCC; continuing medical education = CME; electronic health record = EHR; information systems = IS; information technology = IT; mobile health = mhealth; population = N; quality improvement = QI; process improvement = PI; professional development = PD; root cause analysis = RCA.

| **EVIDENCE and FINDINGS** | **MANIFESTATIONS** | **ANALYSIS** | **INDIVIDUAL USER ADJUSTMENTS** | **ORGANIZATIONAL ADJUSTMENTS** |
| --- | --- | --- | --- | --- |
| **Clinical care** | | | | |
| Engagement and communication problems | Different quality eye contact  Missed simultaneous words  Feels less responsive | Video ≠ in-person  Requires > concentration, fewer distractions | Use an “ice breaker”  Adjust communication (e.g., verbal > tissue for tears)  Fit equipment to task: 1 large or 2 screens | Train on customer service and video etiquette  Schedule real breaks  Provide equipment funds |
| Less rewarding and spontaneous | Less warm, deep and therapeutic for clinician | Less tangible, view limitations and virtual not preferred | Reflect on and implement rewarding aspects (e.g., meaning, joy) | Blend in-person and video schedules  Remunerate well |
| Draining and tiring, > complex and requires effort  -Cognitive  -Emotional  -Physical | Fatigue and poor setting/office, workflow or routine  -Less focus and attention  -Irritability, criticism  -Aches, strain | Evaluate emotional, cognitive, physical and other dimensions  -Individual (e.g., trait or state) and workforce issue(s) | Improve preparation, staff assistance and workflow  -Discourage multi-tasking  -Take breaks and walks  -Tend to ergonomics  -Meditate and use yoga | Provide pre-, para- and post-session assistance  Reduce pre- and post-session workload (e.g., EHR)  Schedule real breaks between sessions |
| Excessive screen time | Eye, wrist, neck, head and other fatigue complaints | Furniture, ergonomic, screen and accessory issues | Adjust ergonomics, eyewear, breaks and length of day | Pre-plan basic needs and customize to individual needs |
| Interruptions | Texts, calls, and other alarms | Allow interruptions if purposeful and necessary | Silence alarms and limit e-mail/text  Pre-notify patients | Use teams for coverage planning |
| Quality of care outcomes incomplete | Compare in-person and video metrics with smaller N in depth | Use established questionnaires  Identify root causes | Request feedback  Ask in-session and make adjustments | Add fatigue assessments into metric panels |
| **EVIDENCE and FINDINGS** | **MANIFESTATIONS** | **ANALYSIS** | **INDIVIDUAL USER ADJUSTMENTS** | **ORGANIZATIONAL ADJUSTMENTS** |
| **Clinical care (Cont)** | | | | |
| Care and other impact metrics may not be meaningful | Not inclusive of adjustments with video, EHR and other | Detect if clinician experience is good and feels effective | Align work to goals  Reconsider how impact shown (e.g., rural access) | Identify which PI/QI metrics work/needed to show impact |
| **Human factors** | | | | |
| Amplification (shift from seldom to much use) | Physical ailments  Fatigue complaints | Minor issues become problems | Identify tedious or problematic workflow context(s) and modify | Watch aggregate trending across settings/services  Individual/team check-ins |
| Cumulative technology load  -Work  -Life | Emotional, cognitive, physical fatigue/tiredness  Put off/delayed events | Recreation (e.g., game), social (media), training and care add up | Identify what needs to be/ in-person and triage/shift  Pre-plan schedules  Get advice | Set aside meeting/rounds time to encourage reflection and feedback  Diversify activity modes |
| Work engagement vs negative or cynical | Negative comments, schedule changes | Trait, state and other causes of fatigue | Work vs. goals alignment  Attitude to self/others | Monitor trust, conflict and worry |
| Fitness (emotional, physical, spiritual) vs fatigue | Concerns, worries, appearance and personal disclosures | Personal/professional, individual/team/ group evaluation | Localize problem and check-in with supervisor  Add good habits | Customize workload  Monitor for burnout  Provide fitness/diet options |
| Isolation vs. social/interpersonal connection | Missing discussions, meetings and shifts  Poor teamwork | Temporary or prolonged  State vs. trait/chronic | Evaluate culture of care, training and faculty development | Monitor for participation/ presence/part of vs distracted/ absent |
| Errors | Missed details, poor outcomes and EHR alerts/alarms | Individual, group, team and IS factor(s) | Identify common errors and get feedback  Review performance data | Provide performance data  QI/PI for input and response with RCA analyses |
| Myth of multi-tasking | Patient survey, errors and observed disengagement | Facilitate reflection, use peer observation or train proactively | Engage/focus on task  Multi-task purposely (e.g., delayed log-on, task shift) | Reward and role model engagement with others  Feedback on errors |
| Effectiveness vs negative perception self/others/resources | Loss of ideals and standards  Frustration and criticism | Care, technology, workflow and social mismatches | Employee-supervisor prioritization, adjust workflow and add help | Monitor WB and precursors to burnout and cynicism |
| **Training and professional development** | | | | |
| Task-specific training and monitoring | Video and EHR easier to generalize | Skills, optimizing experience and flow | Attend training and share tips in CCCs | Simplify workflow with support; ask for input |
| **EVIDENCE and FINDINGS** | **MANIFESTATIONS** | **ANALYSIS** | **INDIVIDUAL USER ADJUSTMENTS** | **ORGANIZATIONAL ADJUSTMENTS** |
| **Training and professional development (Cont)** | | | | |
| Skills/competencies | Asynchronous less routine | Skills, attitude and knowledge, decision-making and triage | Schedule time (rather than free time) | Build into clinical work schedule, remunerate for time % quality |
| Learning online may not be effective as in-person | Convenient, topical and limited discourse  PD acculturation and roles = in-depth | Distractions, multi-tasking interfere  CME protects time and networking good | Identify what needs to be/ in-person: skills + attitudes  Prioritize essential in-person conferences | Centralize virtual vs sessions/courses in-person  Support in-person PD conferences |
| Teaching effectiveness and flow | In-person effective and > spontaneous  Virtual: time needed to shift material, method and evaluation | In-person inefficient with > wing it  Virtual preparation reduces errors and disrupted process | Identify what needs to be/is better in-person and set learner requirement  Set expectation/culture for seminar/rounds | Budget video and accessories according to outcomes  Budget time and remunerate shift to virtual |
| Social and interpersonal relationships | Less connected  Lost networking and venting | > individual and less team  Less collegial, less part of and isolated | Consider meetings really needed  Real meeting before/ after meeting/session | Consider tangibles and intangibles of community  Non-$ rewards = teamwork, relationships and support |
| Administration and committee work | In-person meetings long and dry  Virtual easier | Offer socialization  Good if folks present | Align role to task (e.g., leader early + in-person)  Request video + audio on | Set organizational culture and expectations: engagement and productivity |
| **Workflow and administration** | | | | |
| Extra planning and organization | Battery/WiFi failure  Difficult or time-consuming access | Team/functional approach better than individual/structural | Mobile devices and hotspots facilitate and optimize workflow | Re-assess needs/resources  Add clerical and technical support |
| Technology dependence creates workflow labor | EHR retrieving, analyzing and documenting | Difficult for clinician, better for overall system | Back-up systems, ease of access and other assistance | Provide pre-, para-and post-session assistance |
| Higher % time at bottom of license  Video and EHR adds up | EHR: fields, checklists and other; many not best for MD to do | EHR demand outweighs user needs and rewards | Adjust workload and provide rewards  Shift workload to team | Team-based care model to share workload  Use Lean processes |
| Mis-aligned workflow across settings | Clinicians need office, mobile and home | WiFi, VPN and mobile device use and ease | Assess workflow for settings, request and triage | Assess and pilot telework via checklist and video analysis |
| **EVIDENCE and FINDINGS** | **MANIFESTATIONS** | **ANALYSIS** | **INDIVIDUAL USER ADJUSTMENTS** | **ORGANIZATIONAL ADJUSTMENTS** |
| **Workflow and administration (Cont)** | | | | |
| Productivity, space and efficiency issues | Less efficient and necessary vs detail extraneous | Technology and task fit to license, workflow and preparation issues | Identify time sinks, ask for workflow Lean analysis and take on/trade off tasks | Budget stratify sinks  Re-invest space $ to staff |
| Technology failure(s) reported | Battery/WiFi  Hardware/software  Log-on/other delays | Technology and task fit to workflow, pilot/ preparation and other | Pilot, log and feed problem to IT staff and administration/IS | Pilots and Lean processes  User-centered design studio for new builds |
| EHR and IS network complexity and workload | Time, detail and effort requirements  -Inbox  -Flags/alarms  -E-consult/mhealth  -Fields, checklists | Before, in and after session  Timeliness initial response + ongoing  Completion at work vs. home | Assess workload, obtain skills and become efficient  Request assistance  Discuss with supervisor, mentor and administration | Use occupational health, design studio and PI steps  Monitor precursors to burnout, ineffectiveness and cynicism |
