# Supplementary material for: Findings and Guidelines on Provider Technology, Fatigue, and Well-being: Scoping Review
Source: J Med Internet Res. 2022 May 25;24(5):e34451. doi: 10.2196/34451 (PMC9178447; doi:10.2196/34451)
Supplement: Multimedia Appendix 4 [file jmir_v24i5e34451_app4.docx]

**TABLE 4. GUIDELINES FOR CLINICIANS, SYSTEMS AND ORGANIZATIONS IN HEALTH CARE FOR TECHNOLOGY USE AND WELL-BEING.**

- Shift to culture of well-being with technology use:
  - Technology-, physical environment-, occupational health- and other-specific evaluations and interventions for cognitive, behavioral, emotional and physical dimensions
  - Objective measures to promote/enhance well-being
  - Link well-being, quality of care and other health system metrics
- Individual, job/workplace (e.g., clinic, hospital), system/organizational levels
  - Individuals:
    - Change work patterns (e.g., work less, take more breaks, life-work balance; develop coping skills (e.g., cognitive restructuring, conflict resolution, time management); obtain family/colleague social support; utilize relaxation, mindfulness and fitness strategies [24-25].
    - Learn from those driving technological advancement and help with integration (but may be more personal than professional [2,50].
    - Competencies for: video (2015, 2018), social media (2018), mobile health (2019, 2020) and asynchronous technologies [2,37,44].
  - Job/workplace: redesign job tasks; improve recognition notable individual/team work; use accurate and meaningful performance data; develop fair and equitable policies.
  - Organizational: for prevention/alleviation fatigue/burnout use 360-degree perspective of functioning for well-being related to technological workflow integration.
- Clinical workflow:
  - Evaluate: therapeutic alliance, quality care, access and other parameters from clinician’s point-of-view.
  - Evaluate time spent for each technology, in general, and for tasks thereof, during and after hours (e.g., EHR) to set appropriate limits, taking breaks and/or adjusting other workflow conditions
  - Quantitative and qualitative analysis of each technology to see associated factors, types of fatigue and patterns to guide prevention and amelioration efforts
  - Employ user design approaches/studios with focus groups, studies, surveys [2,85,89], with: better device visuals; (more) immediate chat or in time help options; ease of use; ease of learning; quality of support information event errors [6-7,30,52].
- Institutional strategies and components of a framework
  - Explore impact of health, fatigue and burnout – as well as well-being – at the team, unit and department level
  - Identify approach and responsible parties within information systems (IS, information technology (IT) and other departments
  - Provide leadership, organizational science and resources needed to promote resilience and self-care, ﬂexibility, autonomy, cultivate camaraderie and teamwork [29,51,83].
  - Adapt video and asynchronous technology competencies
    - Core areas (5): Patient-Centered Care; Evaluation/Outcomes; Training/Education; Teams, Professions and Systems within Institutions; Institutional/Organizational Culture [2,74].
    - Additional areas (2): Professional Development and Well-being to develop skills and shift culture [7,29,49-51].
  - Employ strategies to prevent technology-associated fatigue:
    - Include *work, social and* *interpersonal* factors on spectrum with *individual, personal and home* factors plus dimensions and levels above.
    - Organize challenges by how manifest to inform individual and organizational adjustments for prevention (see Table 2).
    - Apply adjustments to telework from home or mobile care.
  - Shift from start-up, cross-sectional approaches based on education without user-centered designs to longitudinal interventions and process improvement across health system [53,56].
  - Build on AMIA usability and human factors recommendations for health IT, research (e.g., standardized use cases, core measures for adverse events), policy (e.g., standardization, interoperability and accounting with adverse event reporting system), industry (e.g., user interface style guides, usability assessments for safety) and clinical end-users (e.g., best practices for implementation and ongoing management) [53,56].
  - Use Lean methods, interprofessional education, team-based care, organizational approaches to well-being (e.g., Ofﬁce of Staff Services) [2,37,51].
- Organizational and community approaches for well-being to extend and/or make specific to technology-related integration and workflow:
  - American Medical Association (AMA): establish wellness as quality indicator, initiate selected interventions, reﬁne over time [23].
  - AMA and others: start wellness committee/choose wellness champion, distribute annual wellness survey to assess/monitor, meet regularly with participants to discuss data and interventions [2,29,51].
  - Mayo Clinic’s and Institute for Healthcare Improvement’s “Improving Joy in Work”: acknowledge and assess problem; develop and implement targeted interventions; use rewards/incentives to facilitate meaning and purpose; align values and strengthen culture; measure in real-time [29,51,83].
  - Joy in Practice Framework affirms need to address human needs/factors, develop leaders’ participative management competency [28].
- Organizational and community approaches on EHR usability that need to assess fatigue:
  - Healthcare Information Management and Systems Society defines usability attributes: simplicity, natural-ness, consistency, forgiveness and feedback, effective use language, efﬁcient interactions, effective information presentation, preservation of context, minimization cognitive load.
  - Expansion includes 14 EHR usability principles and effective user-centered design (UCD); usability testing and evaluation; post-deployment monitoring and patient safety [53,56].
  - Challenges: complexity EHR interaction within full socio-technical context; professional roles intended users; peculiarities clinical collaboration; measuring downstream inﬂuences of systems.
